# The clinical value of pharmacogenomics in the pharmacotherapy of common psychiatric disorders: a macroscopic analysis based on real-world data

**DOI:** 10.1186/s12888-026-08198-4

**Published:** 2026-05-23

**Authors:** Fei Jia, Yanjie Zhao, Shanshan Tian, Nan Wang, Xiaoqian Lan, Mengxi Niu, Shuang Bao, Yannan Zang, Linghui Meng, Pengfei Li, Gang Wang

**Affiliations:** 1 Beijing Key Laboratory of Mental Disorders, National Clinical Research Center for Mental Disorders & National Center for Mental Disorders, Beijing Anding Hospital, Capital Medical University, Beijing, 100088 China; 2 Beijing Key Laboratory of Critical Bridging Technologies for Chronic Disease Drug Development, Beijing, 100088 China

**Keywords:** Pharmacogenomic testing, Schizophrenia, Depressive disorder, Bipolar disorder, Real-world, Macroscopic analysis

## Abstract

**Background:**

Pharmacogenomic (PGx) testing holds promise for personalized psychiatry, but its clinical utility in real-world inpatient settings remains inadequately established. Using real-world data, this study investigated whether PGx testing is associated with improved treatment outcomes in hospitalized patients with schizophrenia (SCZ), major depressive disorder (MDD), and bipolar disorder (BD).

**Methods:**

A retrospective cohort study was conducted, enrolling inpatients with SCZ, MDD, and BD from January 2022 to March 2024. Patients were divided into a PGx group or a treatment-as-usual (TAU) group based on whether they received PGx testing. The primary outcomes were disease-specific scale scores (PANSS for SCZ; HAMD/HAMA for MDD; YMRS/HAMD for BD). Demographic characteristics, length of hospital stay, and hospitalization costs were also compared between the two groups.

**Results:**

In total, 3,942 patients were included (SCZ: 1,328; MDD: 1,143; BD: 1,471). Linear mixed model analyses revealed significant negative interaction effects between PGx testing and treatment duration across all three disorders (SCZ-PANSS: -0.86, *p* < 0.001; MDD-HAMD: -0.49, *p* < 0.001; MDD-HAMA: -0.32, *p* < 0.001; BD-YMRS: -0.25, *p* = 0.025; BD-HAMD: -0.39, *p* < 0.001). This indicates that symptom improvement over time was significantly faster in the PGx group. Furthermore, for MDD and BD, the PGx group had more severe baseline symptoms (MDD: HAMD score 2.77 points higher, *p* < 0.001; HAMA score 1.79 points higher, *p* < 0.001. BD: HAMD score 2.16 points higher, *p* < 0.001) and higher hospitalization costs (MDD: ¥8,730.06 higher, *p* < 0.001; BD: ¥2,902.78 higher, *p* = 0.025). Additionally, MDD patients in the PGx group had a significantly longer hospital stay (mean difference: 4.25 days, *p* = 0.02).

**Conclusion:**

In the real-world inpatient setting, PGx testing was associated with a steeper slope of clinical symptom improvement in patients with SCZ, MDD, and BD. It is important to note that PGx testing was used more often for patients with more severe baseline conditions who also required more healthcare resources. Nevertheless, PGx may help optimize treatment regimens and enhance therapeutic efficiency. Overall, these findings provide practical evidence supporting PGx as a useful clinical decision-support tool for hospitalized psychiatric patients.

**Clinical trial number:**

Not applicable. This study is a retrospective observational study using real-world clinical data and did not involve a prospective intervention requiring trial registration.

**Supplementary Information:**

The online version contains supplementary material available at 10.1186/s12888-026-08198-4.

## Introduction

Pharmacogenomics (PGx) examines how genetic variation affects an individual’s responses to drugs [1]. It primarily explores the impact of genetic polymorphisms encoding drug-metabolizing enzymes, drug transporters, and drug targets on pharmacokinetics and pharmacodynamics. The clinical application of pharmacogenomics may support the implementation of individualized dosing and precision medicine, thereby potentially improving the level of clinical pharmacotherapy. Currently, PGx biomarker testing is recommended in over 600 medication labels by the U.S. Food and Drug Administration (FDA); anticancer, neurological, and psychiatric drugs constitute the largest part of the top 3 categories [2]. Since 2005, the FDA has begun to issue guidelines related to PGx. Up to now, there have been multiple platforms dedicated to the research and knowledge sharing of PGx [3–6]. In the case of psychiatry, significantly robust evidence suggests that changes in drug dosages or early warnings of adverse reactions can be achieved by analyzing the polymorphisms of genes such as *CYP2D6*, *CYP2C19*, *HLA*, and *HTR2A* [7]. Likewise, the Clinical Pharmacogenetics Implementation Consortium (CPIC) has also published corresponding documents [8–10].

To date, randomized controlled trials (RCTs) of pharmacogenomics-guided pharmacotherapy for psychiatric disorders have yielded inconsistent results [11, 12]. Previous trials have been limited by high risk of bias, small sample sizes [13], and population heterogeneity [14], which reduce their methodological strength. A meta-analysis even casts doubt on the usefulness of the currently available PGx tools in the treatment of depression [15]. Moreover, PGx tests and clinical decision-support tools are not yet standardized, leading to result interpretation and prescribing recommendations that are variable [16]. A survey across medical specialties found that even psychiatrists remain skeptical about the benefits of PGx testing [17]. Hence, it is both important and clinically meaningful to carry out a macroscopic analysis based on real-world data to assess the efficiency of PGx and its determinants in psychiatric clinical ‍‌practice.

This‌ study selected three prevalent psychiatric disorders—schizophrenia (SCZ), major depressive disorder (MDD), and bipolar disorder (BD)—as representative conditions. We assessed the influence of PGx testing on treatment effectiveness, length of hospital stay, and hospitalization costs to investigate the clinical benefits of PGx in a real-world setting at a macroscopic level. We also aimed to provide evidence to inform the rational use of PGx technology in the Chinese population.

## Materials and methods

### Materials

Genomic DNA from the peripheral blood of all participants of the study was isolated. Pharmacogenomic testing was carried out using the Ex-DNA Whole Blood Genomic Nucleic Acid Extraction or Purification Kit (Xi’an Tianlong Technology Co., Ltd.). The profiling was done by matrix-assisted laser desorption/ionization time-of-flight mass spectrometry (MALDI-TOF MS) on the DP-TOF Mass Spectrometry System (Zhejiang Dipo Diagnostic Technology Co., Ltd., SNP platform). All samples were processed according to the manufacturer’s protocols. Experimental operations were performed at the Clinical Gene Testing Laboratory of Beijing Anding Hospital, Capital Medical University. This laboratory participates in the annual external quality assessment program organized by the National Health Commission of China for *CYP2C19*, *CYP3A5*, and *CYP2D6* genotyping, and consistently achieves passing scores. This guarantees the standardization and quality control of the testing ‍‌process.

### Study design and data sources

This was a retrospective cohort study. All patients who met the inclusion criteria during the study period were included. Basic data and disease severity scores of patients admitted to our hospital from January 1, 2022, to March 31, 2024, were obtained through the Hospital Information System (HIS). Depending on whether they had undergone PGx testing, patients were assigned either to the Pharmacogenomic Testing (PGx) group or the Treatment-as-Usual (TAU) ‍‌group.

For patients who underwent PGx testing, only the index admission during which PGx testing was performed was included (including tests conducted in either outpatient or inpatient settings). For patients in the TAU group, only the first hospitalization during the study period was included. If a patient had multiple hospitalizations, only the first eligible admission was retained to avoid clustering effects and to ensure independence of observations.

No age restrictions were applied at the inclusion stage. The actual age range of the included population was 9 to 82 years (see Section “Inclusion and exclusion criteria”). The mean age is reported in the baseline characteristics (Tables 1, 4 and 7), and age-stratified subgroup analyses are available in the Supplementary Material.

Analyses were conducted separately by disease diagnosis. For each diagnosis, we compared socio-demographic characteristics, treatment effectiveness, length of hospital stay, and hospitalization costs between the PGx and TAU groups. In our clinical setting, PGx testing was typically ordered for patients with complex or treatment-resistant conditions, either during the early phase of hospitalization or after a failed medication trial. The timing of testing was at the discretion of the treating physicians based on clinical judgment.

PGx test results were presented in a structured laboratory report. The report included gene–drug interaction classifications, covering metabolic genes, efficacy-related genes, and adverse reaction-related genes. These reports were provided to clinicians as a reference tool. This study did not systematically document whether or how prescribing decisions were adjusted based on the PGx results. Therefore, PGx results should be viewed as one of several factors considered alongside other clinical information, rather than as a standalone driver of treatment decisions.

### Inclusion and exclusion criteria

Inclusion Criteria: (a) Hospitalized patients, with no restrictions on age or gender. The actual age range of the included patients was 9–82 years. (b) Meeting the diagnostic criteria for schizophrenia, depressive disorder, or bipolar affective disorder according to International Classification of Diseases, 10th Revision (ICD-10) (with group comparisons performed by disease type). (c) Documentation in the medical record of at least two assessments using disease-specific rating scales (Positive and Negative Syndrome Scale (PANSS) [18] for schizophrenia; Hamilton Depression Rating Scale (HAMD) [19] for depressive disorder; Young Mania Rating Scale (YMRS) [20] and HAMD for bipolar affective disorder).

Exclusion Criteria: (a) Non-hospitalized patients. (b) Patients not meeting the diagnostic criteria for schizophrenia, depressive disorder, or bipolar affective disorder, or having other clinical diagnoses. (c) Patients with significant liver disease, a history of chronic liver pathology, abnormal liver function tests, renal insufficiency, or severe cardiovascular, pulmonary, hematological, endocrine, or central nervous system diseases; patients with malignant tumors. (d) Patients for whom disease-specific rating scale scores were unavailable or who had only one recorded scale score.

### Outcome measures

The following outcome measures were assessed in this study:

Symptom severity improvement: For each disease-specific scale (SCZ: PANSS; MDD: HAMD and Hamilton Anxiety Rating Scale (HAMA) [21]; BD: YMRS and HAMD), symptom improvement was evaluated using:

Absolute reduction: admission score minus discharge score.

Percentage reduction: (admission score – discharge score) / admission score × 100%.

Symptom scales were assessed weekly during hospitalization as part of routine clinical care. For each patient, the first and last available scale scores during the index admission were used for analysis.

The rate of symptom improvement over time was assessed using linear mixed models. The interaction term between PGx testing and time (measured in weeks of hospitalization) served as the primary indicator of differences in improvement trajectories between the PGx and TAU groups.

Length of hospital stay: defined as the total number of days from admission to discharge.

Hospitalization costs were defined as the total direct medical costs incurred during the hospitalization period. These included medication, laboratory tests, bed fees, and other clinical services. The cost of PGx testing was excluded from the analysis to avoid confounding the cost comparison between groups.

### Statistical analysis

Statistical descriptions were presented with means and standard deviations (SD) for continuous variables or frequencies and percentages for categorical variables. Univariable analyses were performed using t tests or Chi-square tests, as appropriate. Standardized mean differences (SMD) between groups were also reported in univariable analyses.

Longitudinal changes in psychiatric symptom outcomes were analyzed using linear mixed-effects models (LMM). Time length of hospital stay (week), where to undergo pharmacogenomic testing (PGx), and their interaction item were included as fixed effects to assess differences in symptom change trajectories between PGx groups. Sex, age, and the use of modified electroconvulsive therapy (MECT) during hospitalization were additionally adjusted as covariates. To account for within-subject correlation arising from repeated measurements, a random intercept was specified for each patient (de-identified hospitalization ID). Model parameters were estimated using restricted maximum likelihood estimation. Fixed-effect estimates are presented with corresponding Wald-based 95% confidence intervals (CIs), statistic z and two-sided p values. All statistical analyses were conducted in R language version 4.5.0 [22].

### Identification and management of biases

This was a retrospective study using data collected from routine clinical practice, and therefore carries inherent limitations associated with this design. PGx testing was not randomly assigned but was rather made on the basis of the clinical judgment of the doctor and the patient’s preference. Hence, there could be selection bias due to patient choice that may have influenced the PGx and TAU cohorts and such bias could be related to patients’ disease severity, history of prior treatments, comorbidities, socioeconomic class (e.g., ability to pay and insurance coverage), and physician prescribing preferences. After adjusting for measurable confounding factors with multivariate models and checking baseline balance, our statistical method can hardly allow for the complete exclusion of the effect of hidden or unmeasured confounding ‍‌factors. Therefore, within the framework of this study design, the observed statistical associations between PGx testing and clinical outcomes are inherently correlational rather than causal, and their interpretation in terms of causal inference is limited.

## Result

### Schizophrenia (SCZ) group

#### Baseline characteristics

A total of 1,328 patients with schizophrenia were included in the analysis. As shown in Table 1, no statistically significant differences were observed between the PGx and TAU groups in terms of age, initial PANSS score, or primary clinical diagnosis (*p* > 0.05). However, the proportion of female patients was significantly higher in the PGx group, approximately 9% greater than in the TAU group (*p* < 0.001). Similarly, the proportion of patients with medical insurance was significantly higher in the PGx group, approximately 10.7% greater than in the TAU group (*p* < 0.001).

**Table 1:** Baseline characteristics of patients with SCZ (*n* = 1328)

Variable	PGx Testing Performed		Univariable Analysis		
	Yes (*n* = 531)	No(*n* = 797)			
	*n* (%)	*n* (%)	Chi-square	*p*	SMD
Gender = Female	369 (69.5)	482 (60.5)	10.86	< 0.001^^^	0.190
Medical Insurance = Yes	319 (60.1)	394 (49.4)	14.09	< 0.001^^^	0.215
Primary Clinical Diagnosis			—	0.47*	0.149
Undifferentiated Schizophrenia	345 (65.0)	538 (67.5)			
Paranoid Schizophrenia	161 (30.3)	217 (27.2)			
Other	25(4.7)	42(5.3)			
	Mean (SD)	Mean (SD)	*t*	*p*	SMD
Age (years)	36.89 (12.45)	35.98 (12.90)	1.28	0.20	0.071
Initial PANSS Score	63.67 (20.44)	64.87 (20.16)	-1.06	0.29	0.059

*Fisher’s Exact Test, ^*p* < 0.05, ^^*p* < 0.01, ^^^*p* < 0.001, indicating a statistically significant difference

#### Clinical outcomes

As presented in Table 2, the final PANSS scores were significantly lower in the PGx group compared to the TAU group (*p* < 0.001), and the PANSS reduction percentage was higher in the PGx group (*p* = 0.043). No statistically significant differences were found between the two groups in terms of PANSS reduction score, length of hospital stay, or hospitalization costs (*p* > 0.05).

**Table 2:** Clinical outcomes of patients with SCZ (*n* = 1328)

Variable	PGx Testing Performed		Univariable Analysis		
	Yes (*n* = 531)	No(*n* = 797)			
	Mean (SD)	Mean (SD)	t	*p*	SMD
Length of Hospital Stay (days)	45.20 (30.89)	43.85 (50.75)	0.60	0.55	0.032
Final PANSS Score	45.72 (13.19)	48.39 (14.26)	-3.46	< 0.001^^^	0.194
PANSS Reduction Score	17.93 (18.02)	16.49 (17.06)	1.45	0.15	0.082
PANSS Reduction Percentage (%)	24.30 (22.11)	21.59 (25.87)	2.03	0.043^	0.113
Hospitalization Cost (CNY)	56745.51 (32055.59)	52944.23 (42761.03)	1.85	0.065	0.101

#### Linear mixed model analysis results

The linear mixed model analysis results (Table 3) showed that weeks of hospitalization had a significant negative effect on PANSS scores (-0.98, *p* < 0.001). The main effect of whether PGx testing was performed was not significant (*p* = 0.32), but its interaction with time was significantly negative (-0.86, *p* < 0.001). This indicates that although baseline symptoms were similar between the two groups, the PGx group experienced a faster rate of symptom improvement during treatment.

**Table 3:** Linear mixed model analysis of score changes with PGx treatment in patients with SCZ†

Variable	Estimate	UL	LL	statistic	*p*
Weeks of Hospitalization	-0.98	-1.11	-0.85	-14.60	< 0.001^^^
PGx Testing Performed (Yes vs. No)	0.95	-0.94	2.84	0.99	0.32
Weeks of Hospitalization × PGx Testing Performed (Yes vs. No)	-0.86	-1.10	-0.61	-6.85	< 0.001^^^

†The model adjusted for sex, age, and MECT as covariates. Detailed results are available in the supplementary material

### Major depressive disorder (MDD) group

#### Baseline characteristics

A total of 1,143 patients with major depressive disorder were included in the analysis. As shown in Table 4, no statistically significant differences were observed between the PGx and TAU groups in terms of gender, medical insurance coverage, age, or primary clinical diagnosis (*p* > 0.05). However, the initial HAMD and HAMA scores were significantly higher in the PGx group compared to the TAU group (*p* = 0.001 and *p* = 0.009, respectively), indicating greater baseline symptom severity in the PGx group.

**Table 4:** Baseline characteristics of patients with MDD (*n* = 1143)

Variable	PGx Testing Performed		Univariable Analysis		
	Yes (*n* = 515)	No (*n* = 628)			
	*n* (%)	*n* (%)	Chi-square	*p*	SMD
Gender = Female	410 (79.6)	478 (76.1)	1.80	0.18	0.084
Medical Insurance = Yes	250 (48.5)	271 (43.2)	3.10	0.078	0.108
Primary Clinical Diagnosis			—	0.28*	0.222
Recurrent depressive disorder, current episode severe without psychotic symptoms	316 (61.4)	374 (59.6)			
Recurrent depressive disorder, current episode severe with psychotic symptoms	86 (16.7)	121 (19.3)			
Recurrent depressive disorder, current episode moderate with somatic symptoms	51 (9.9)	50 (8.0)			
Other	62(12.0)	83(13.2)			
	Mean (SD)	Mean (SD)	*t*	*p*	SMD
Age (years)	39.72 (18.58)	39.29 (18.05)	0.39	0.69	0.023
Initial HAMD Score	22.78 (10.48)	20.73 (10.89)	3.23	0.001^^	0.192
Initial HAMA Score	15.76 (9.05)	14.33 (9.26)	2.62	0.009^^	0.156

#### Clinical outcomes

As presented in Table 5, the reduction scores for both HAMD and HAMA were significantly greater in the PGx group compared to the TAU group (*p* = 0.004 and *p* = 0.006, respectively). The HAMD reduction percentage was also significantly higher in the PGx group (*p* = 0.016), while the HAMA reduction percentage did not differ significantly between groups (*p* = 0.34). The length of hospital stay was approximately 4.25 days longer in the PGx group than in the TAU group (*p* = 0.002), and hospitalization costs were ¥8,730.06 higher in the PGx group (*p* < 0.001).

**Table 5:** Clinical outcomes of patients with MDD (*n* = 1143)

Variable	PGx Testing Performed		Univariable Analysis		
	Yes (*n* = 515)	No (*n* = 628)			
	Mean (SD)	Mean (SD)	t	*p*	SMD
Length of Hospital Stay (days)	33.98 (21.40)	29.73 (25.28)	3.08	0.002^^	0.182
Final HAMD Score	7.47 (8.04)	7.34 (7.47)	0.29	0.77	0.017
HAMD Reduction Score	15.35 (11.01)	13.41 (11.32)	2.91	0.004^^	0.173
HAMD Reduction Percentage (%)	63.95 (33.77)	56.04 (71.94)	2.42	0.016^^	0.141
Final HAMA Score	5.43 (6.13)	5.40 (6.04)	0.08	0.94	0.005
HAMA Reduction Score	10.39 (9.18)	8.93 (8.56)	2.74	0.006^^	0.164
HAMA Reduction Percentage (%)	59.25 (50.09)	56.50 (44.38)	0.95	0.34	0.058
Hospitalization Cost (CNY)	47879.87 (30596.43)	39149.81 (26073.03)	5.13	< 0.001^^^	0.307

#### Linear mixed model analysis results

The linear mixed model analysis results (Table 6) indicated that Weeks of Hospitalization had a significant negative effect on both HAMD and HAMA scores (Estimate = -1.76 and − 1.21, respectively, *p* < 0.001), demonstrating that both depressive and anxiety symptoms decreased over the course of hospitalization. Baseline scores for both scales were significantly higher in the PGx group (HAMD: 2.77, *p* < 0.001; HAMA: 1.79, *p* < 0.001), suggesting greater initial symptom severity in this group. The interaction term between PGx testing and time was significantly negative (HAMD: -0.49, *p* < 0.001; HAMA: -0.32, *p* < 0.001), indicating a faster rate of symptom improvement in the PGx group.

**Table 6:** Linear mixed model analysis of score changes with PGx treatment in patients with MDD

Scale	Variable	Estimate	UL	LL	statistic	*p*
HAMD Score	Weeks of Hospitalization	-1.76	-1.92	-1.60	-22.00	< 0.001^^^
	PGx Testing Performed (Yes vs. No)	2.77	1.71	3.84	5.12	< 0.001^^^
	Weeks of Hospitalization × PGx Testing Performed (Yes vs. No)	-0.49	-0.73	-0.26	-4.13	< 0.001^^^
HAMA Score	Weeks of Hospitalization	-1.21	-1.33	-1.08	-19.20	< 0.001^^^
	PGx Testing Performed (Yes vs. No)	1.79	0.91	2.70	3.99	< 0.001^^^
	Weeks of Hospitalization × PGx Testing Performed (Yes vs. No)	-0.32	-0.51	-0.14	-3.47	< 0.001^^^

### Bipolar disorder (BD) group

#### Baseline characteristics

A total of 1,471 patients with bipolar disorder were included in the analysis. As shown in Table 7, the proportion of female patients was significantly higher in the PGx group compared to the TAU group (*p* < 0.001), as was the proportion of patients with medical insurance (*p* < 0.001). No significant differences were observed in age or primary clinical diagnosis (*p* > 0.05). For symptom severity, no significant difference was found in initial YMRS scores between the two groups (*p* = 0.81). However, the initial HAMD scores were significantly higher in the PGx group (*p* = 0.001), indicating greater baseline depressive symptom severity in this group.

**Table 7:** Baseline characteristics of patients with BD (*n* = 1471)

Variable	PGx Testing Performed		Univariable Analysis		
	Yes (*n* = 501)	No (*n* = 970)			
	*n* (%)	*n* (%)	Chi-square	*p*	SMD
Gender = Female	381 (76.0)	629 (64.8)	18.75	< 0.001^^^	0.247
Medical Insurance = Yes	307 (61.3)	474 (48.9)	19.94	< 0.001^^^	0.251
Primary Clinical Diagnosis			—	0.33*	0.201
Bipolar affective disorder, current episode manic with psychotic symptoms	157 (31.3)	294 (30.3)			
Bipolar affective disorder, current episode manic without psychotic symptoms	137 (27.3)	303 (31.2)			
Bipolar affective disorder, current episode severe depressive without psychotic symptoms	79 (15.8)	122 (12.6)			
Bipolar affective disorder, current episode mixed	82 (16.4)	134 (13.8)			
Other	46(9.2)	117(12.1)			
	**Mean (SD)**	**Mean (SD)**	***t***	***p***	**SMD**
Age (years)	36.03 (13.50)	35.04 (14.18)	1.31	0.19	0.072
Initial YMRS Score	15.32 (12.67)	15.15 (12.21)	0.24	0.81	0.013
Initial HAMD Score	11.75 (11.99)	9.70 (10.26)	3.24	0.001^^	0.184

#### Clinical outcomes

As presented in Table 8, the reduction in HAMD scores was significantly greater in the PGx group compared to the TAU group (*p* = 0.001), and the HAMD reduction percentage was also significantly higher (*p* = 0.030). For manic symptoms, no significant differences were observed between groups in terms of final YMRS score, YMRS reduction score, or YMRS reduction percentage (*p* > 0.05). Hospitalization costs were approximately ¥2,902.78 higher in the PGx group (*p* = 0.025), while no significant difference was found in length of hospital stay (*p* = 0.45).

**Table 8:** Clinical outcomes of patients with BD (*n* = 1471)

Variable	PGx Testing Performed		Univariable Analysis		
	Yes (*n* = 501)	No (*n* = 970)			
	Mean (SD)	Mean (SD)	t	*p*	SMD
Length of Hospital Stay (days)	35.66 (18.44)	34.81 (23.25)	0.76	0.45	0.04
Final YMRS Score	3.18 (5.57)	3.46 (5.59)	-0.91	0.36	0.051
YMRS Reduction Score	12.09 (12.14)	11.70 (11.76)	0.60	0.55	0.033
YMRS Reduction Percentage (%)	72.51 (53.04)	69.56 (47.70)	0.95	0.34	0.058
Final HAMD Score	3.46 (5.52)	3.19 (4.90)	0.91	0.36	0.051
HAMD Reduction Score	8.27 (10.32)	6.50 (8.76)	3.25	0.001^^	0.185
HAMD Reduction Percentage (%)	64.33 (49.77)	56.30 (71.42)	2.17	0.030^	0.130
Hospitalization Cost (CNY)	48960.99 (21687.36)	46058.21 (26677.20)	2.24	0.025^	0.119

#### Linear mixed model analysis results

Results from the linear mixed model analysis (Table 9) showed that the number of hospital weeks had a significant negative effect on both YMRS and HAMD scores (*p* < 0.001). PGx testing had no significant effect on YMRS scores (*p* = 0.26) but showed a significant positive effect on HAMD scores (estimate = 2.16, *p* < 0.001), suggesting greater baseline severity of depressive symptoms in the PGx group. The interaction between PGx testing and time was significant for both scales (YMRS: -0.25, *p* = 0.025; HAMD: -0.39, *p* < 0.001), indicating again that the PGx group experienced a faster rate of improvement in both depressive and manic-related symptoms.

**Table 9:** Linear mixed model analysis of score changes with PGx treatment in patients with BD

Scale	Variable	Estimate	UL	LL	statistic	*p*
YMRS Score	Weeks of Hospitalization	-1.52	-1.64	-1.40	-24.20	< 0.001^^^
	PGx Testing Performed (Yes vs. No)	0.59	-0.43	1.60	1.13	0.26
	Weeks of Hospitalization × PGx Testing Performed (Yes vs. No)	-0.25	-0.48	-0.03	-2.24	0.025^
HAMD Score	Weeks of Hospitalization	-0.95	-1.05	-0.86	-19.20	< 0.001^^^
	PGx Testing Performed (Yes vs. No)	2.16	1.27	3.04	4.78	< 0.001^^^
	Weeks of Hospitalization × PGx Testing Performed (Yes vs. No)	-0.39	-0.56	-0.22	-4.54	< 0.001^^^

**Fig. 1:**
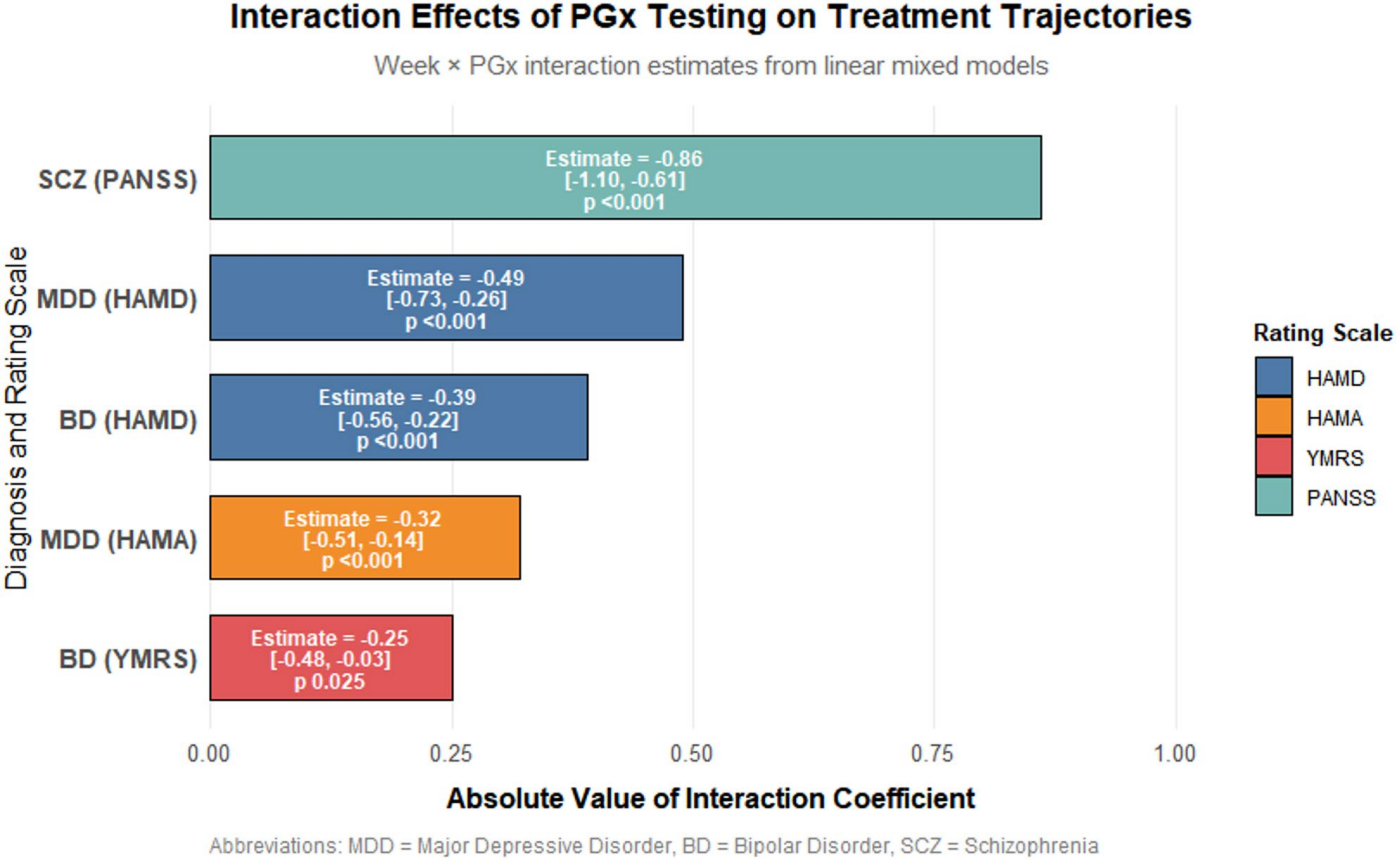
Interaction effects of PGx × Time across different disorders

## Discussion

### Principal findings

This study found that undergoing PGx testing was significantly associated with an accelerated rate of symptom improvement during hospitalization in patients with major depressive disorder (MDD), bipolar disorder (BD), and schizophrenia (SCZ) (Fig. 1). Although the level of evidence is lower than that of RCTs, this macroscopic analysis based on real-world data reflects the overall effect of PGx in routine clinical practice. The patient population included in this macroscopic analysis was highly heterogeneous, encompassing many individuals who may not be the most suitable candidates for PGx (e.g., those without significant drug-gene interactions), which dilutes the potential benefit of PGx for specific subgroups. Therefore, the observed faster symptom improvement in the PGx group likely represents a conservative estimate; the effect may be more pronounced in subgroups with clear pharmacogenetic indications who are more likely to benefit from testing (e.g., individuals with specific metabolic phenotypes or those carrying high-risk genotypes).

#### Analysis of schizophrenia findings in the context of existing literature

In this study, there was no significant difference in baseline PANSS scores between the PGx and TAU groups among patients with schizophrenia. However, the PGx group showed a faster rate of decline in PANSS scores. This finding aligns with the conclusions of two recent RCTs conducted in Chinese populations with schizophrenia [11, 23], which also reported that pharmacogenomics-guided pharmacotherapy showed superior outcomes compared to treatment as usual in terms of both PANSS reduction rate and treatment response rate.

#### Analysis of major depressive disorder findings in the context of existing literature

In this study of patients with major depressive disorder, we found that those who underwent PGx testing experienced a significantly faster rate of improvement in depressive symptoms (HAMD) compared to those who did not. Several studies have reported that PGx may reduce the prescription of medications with high-risk drug-gene interactions [12, 24, 25], increase response and remission rates at weeks 8–12 [26–30], and improve medication adherence while reducing unnecessary medication switches [31]. However, some studies have reported contrasting findings. For instance, an RCT by Oslin et al. [12] showed that although PGx reduced the use of medications with high interaction risks, there was no statistical difference in remission rates at week 24 compared to the treatment-as-usual group. Meta-analyses suggest that the short-term advantages of PGx may diminish over time [32, 33]. The acceleration effect of PGx may be more pronounced in specific clinical subgroups, such as patients with treatment-resistant depression [13, 30, 34, 35].

#### Analysis of bipolar disorder findings in the context of existing literature

In patients with bipolar disorder, PGx testing was significantly associated with faster improvement in depressive symptoms (HAMD). In contrast, its association with manic symptoms (YMRS) was weaker: although an improving trend was observed, the effect size was smaller and did not reach a comparable level of statistical significance. This pattern is consistent with prior reports [36]. This result may be attributable to differences in the PGx evidence base across medication classes. Genetic targets associated with mood stabilizers (e.g., lithium [37], valproate [38]) remain controversial and have not been sufficiently translated into clinical practice [39–41], while the research foundation for using PGx to assess the risk of antidepressant-induced mania is relatively weak [42, 43]. Pharmacogenomics‍‌ may provide several practical applications for bipolar disorder (BD) management. For example, they may assist clinicians in selecting an antidepressant regimen with the lowest risk of triggering a manic episode during the depressive phase [44]. Also, PGx may offer information for dose adjustment when using particular mood stabilizers such as valproate [38]. Additionally, in cases where there is resistance to treatment, it may serve as a source of useful biological data to a broad treatment plan, thus enhancing the overall treatment strategy‍‌ [13].

#### Analysis of baseline imbalances in MDD and BD cohorts

In the MDD and BD groups, the PGx group had more severe initial scale scores and higher hospitalization costs, suggesting the presence of selection bias: clinicians may have been more likely to order PGx testing for more complex or treatment-resistant patients. Such patients, by their nature, have greater healthcare needs and, therefore, lead to higher medical resource consumption [24, 45], rather than PGx testing being the main reason for an increase in healthcare utilization. However, the PGx group still showed faster symptom relief. One possible explanation for this is that PGx test information may improve the accuracy and effectiveness of pharmacotherapy, which could in turn enhance patient medication adherence and reduce unnecessary medication changes due to insufficient efficacy or side effects‌ [46, 47]. Current evidence suggests that the value of PGx lies in assisting physicians, particularly when managing complex or treatment-resistant cases, to develop safer and potentially faster-acting treatment regimens [48, 49], which may shorten the time to achieve effective treatment.

#### Causal inference and residual confounding

Given the observational design of this study, the findings should be interpreted as associations rather than causal effects. Although linear mixed models were used to adjust for measured confounders including sex, age, and MECT use, the possibility of residual confounding cannot be excluded. Specifically, the observed faster symptom improvement in the PGx group may be partly attributable to confounding by indication: clinicians were more likely to order PGx testing for patients with greater baseline severity, more complex clinical profiles, or prior treatment resistance. This pattern, which reflects real-world clinical practice, complicates the direct comparison between groups. Despite statistical adjustment, unmeasured factors—such as physician prescribing preferences, closer clinical monitoring, or higher treatment adherence in the PGx group—may have contributed to the observed differences. Therefore, while this study provides valuable real-world evidence supporting the clinical utility of PGx, causal conclusions cannot be drawn from these data.

### Age-related differences

Age-stratified subgroup analysis (Supplementary Material) indicated that the superior rate of symptom improvement in the PGx group compared to the TAU group showed a clear age-dependent pattern. This advantage was most pronounced in adults aged 18–64 years, whereas it was not significant in adolescent (< 18 years) or elderly (> 65 years) patients. Findings from two studies focusing on adolescent populations [50, 51] indicated that PGx-guided treatment did not demonstrate superior efficacy compared to usual care in this group. Similarly, an RCT [52] found no cognitive benefit from pharmacogenomic guidance in elderly patients. Therefore, we suggest that PGx testing should be prioritised in adult patients over other age groups.

### Socio-demographic characteristics

The conclusions regarding the cost-effectiveness of PGx remain controversial [14, 53–55]. Due to its relatively high cost and the long-term validity of its results, pharmacogenomic testing tends to have higher acceptance among younger patient populations. A survey [56] indicated a higher rate of PGx uptake among female patients, which aligns with the observations in this study. We also found that the proportion of patients with medical insurance coverage was higher in the PGx group compared to the TAU group, and that the PGx group had higher rates of rehospitalization and revisits. This pattern may be attributed to patients with better economic status having greater access to healthcare resources, more consistent opportunities for medical consultation, and higher treatment adherence, rather than indicating poorer clinical outcomes in this group. Therefore, while the higher costs and longer hospital stays observed in the PGx group (particularly in MDD) are likely attributable to greater baseline complexity rather than PGx testing itself, whether PGx improves treatment efficiency or is simply used in already complex and costly patients remains unclear. The impact of PGx on cost-effectiveness therefore remains uncertain and warrants further investigation.

### Clinical applicability and target population

The present findings suggest that PGx testing was preferentially used in patients with greater baseline severity and higher healthcare resource utilization. This pattern is consistent with real-world clinical decision-making, where testing is often reserved for more complex or treatment-resistant cases. Given that these patients nonetheless demonstrated faster symptom improvement, our results support the potential value of PGx in this subgroup. However, whether PGx confers similar benefits in first-episode patients, those with mild-to-moderate illness, or as a routine screening tool remains unclear. Therefore, based on the available data, we suggest that PGx testing may be more appropriately implemented as a targeted strategy for patients with greater clinical complexity or prior treatment resistance, rather than as a universal screening approach. Future prospective studies are needed to clarify the utility of PGx across different clinical subgroups.

## Limitations and future perspectives

This study was conducted at a single center. As our hospital is a National Depression Diagnosis and Treatment Center, it admits a higher proportion of treatment-resistant cases, which may limit the generalizability of the findings. The conclusions might differ in populations with first-episode or mild illness. Therefore, the findings are most directly applicable to complex or treatment-resistant inpatients in specialized psychiatric settings. Their generalizability to outpatient settings, early-stage illness, or general psychiatric populations remains to be established. Furthermore, we were unable to obtain sufficient systematic data on adverse drug reactions; therefore, the impact of PGx on treatment safety could not be assessed.

Large-sample real-world data at the macroscopic level can reveal general trends in clinical practice. Nonetheless, the evidence level of this kind of data is lower as compared to that of the prospective RCTs. Numerous barriers hinder the clinical implementation of PGx, including lack of standardisation in test reports, difficulty integrating results into electronic health record systems, and variability in clinicians’ ability to interpret findings. These issues have not been discussed in this paper. Such elements may affect the efficient translation of PGx into clinical practice [14, 57]. In order to extensively adopt this promising technology [58], there should be more reliable evidence on its utility and cost-effectiveness [53, 59].

To minimize bias and even validate the worth of PGx in the general population, including special categories such as the elderly and adolescents, future studies should be prospective and multi-center in nature [60, 61]. Additionally, it is necessary to conduct a thorough assessment of its long-term safety and the healthcare economic advantages. At the same time, efforts should be made to translate PGx into clinical practice by solving implementation problems, such as physician training and report integration [57, 62], so that PGx can be converted into a more accessible, individualized clinical decision-support ‍‌system.

Furthermore, while this study demonstrated statistically significant associations between PGx testing and faster symptom improvement, the clinical magnitude of this benefit remains less clear. The estimated interaction coefficients (SCZ-PANSS: -0.86 per week; MDD-HAMD: -0.49 per week; BD-YMRS: -0.25 per week; BD-HAMD: -0.39 per week) represent incremental improvements of approximately 0.25 to 0.86 points per week on each scale. Whether these differences translate into clinically meaningful outcomes—such as earlier remission, fewer medication changes, or better tolerability—could not be determined from the available data. Future studies with predefined clinical endpoints are needed to better quantify the clinical relevance of PGx-guided treatment.

## Conclusion

This real-world study of inpatients demonstrated that pharmacogenomic testing is significantly associated with symptom improvement at a faster rate in patients with schizophrenia, major depressive disorder, and bipolar disorder. Although the PGx group had more severe baseline symptoms, they nonetheless showed greater treatment efficiency, possibly by facilitating earlier optimization of drug selection and reducing trial-and-error prescribing. It is important to note that the observed improvement may reflect not only PGx testing itself but also more intensive clinical management and closer monitoring in this group. These findings suggest that PGx may serve as a useful clinical decision-support tool in psychiatry, particularly for adult inpatients. Further studies are needed to determine whether the benefits are more pronounced in specific clinical contexts, such as acute-phase or early-intervention settings.

## Electronic Supplementary Material

Below is the link to the electronic supplementary material.

Supplementary Material 1

## Data Availability

The datasets generated and/or analyzed during the current study are not publicly available due to privacy restrictions but are available from the corresponding author on reasonable request.

## Abbreviations

PGx: Pharmacogenomics
FDA: Food and Drug Administration
CPIC: Clinical Pharmacogenetics Implementation Consortium
SCZ: Schizophrenia
MDD: Major Depressive Disorder
BD: Bipolar Disorder
HIS: Hospital Information System
TAU: Treatment-as-Usual
ICD-10: International Classification of Diseases, 10th Revision
PANSS: Positive and Negative Syndrome Scale
HAMD: Hamilton Depression Rating Scale
YMRS: Young Mania Rating Scale
HAMA: Hamilton Anxiety Rating Scale
SD: Standard Deviations
SMD: Standardized Mean Differences
LMM: Linear Mixed-effects Models
MECT: Modified Electroconvulsive Therapy
CIs: confidence intervals
RCTs: Randomized Controlled Trials
