# Supplementary material for: The clinical value of pharmacogenomics in the pharmacotherapy of common psychiatric disorders: a macroscopic analysis based on real-world data
Source: BMC Psychiatry. 2026 May 23;26:528. doi: 10.1186/s12888-026-08198-4 (PMC13352744; doi:10.1186/s12888-026-08198-4)
Supplement: Supplementary file 1 — Supplementary Material 1 [file 12888_2026_8198_MOESM1_ESM.docx]

**Table 1. Demographic and Clinical Characteristics of Patients with SCZ**

| Variable | Overall  (n=1328) | PGx Testing Performed | | Univariable Analysis | | |
| --- | --- | --- | --- | --- | --- | --- |
|  |  | Yes(n=531) | No (n=797) |  |  |  |
|  | n (%) | n (%) | n (%) | Chi-square | *p* | SMD |
| Gender = Female | 851 (64.1) | 369 (69.5) | 482 (60.5) | 10.86 | <0.001 | 0.190 |
| Medical Insurance = Yes | 713 (53.7) | 319 (60.1) | 394 (49.4) | 14.09 | <0.001 | 0.215 |
| MECT Performed = Yes | 794 (59.8) | 321 (60.5) | 473 (59.3) | 0.12 | 0.73 | 0.023 |
| Primary Clinical Diagnosis |  |  |  | — | 0.47* | 0.149 |
| Acute psychotic disorder with schizophrenic symptoms | 1 (0.1) | 0 (0.0) | 1 (0.1) |  |  |  |
| Simple schizophrenia | 2 (0.2) | 1 (0.2) | 1 (0.1) |  |  |  |
| Catatonic schizophrenia | 1 (0.1) | 0 (0.0) | 1 (0.1) |  |  |  |
| Schizophrenia | 35 (2.6) | 14 (2.6) | 21 (2.6) |  |  |  |
| Post-schizophrenic depression | 9 (0.7) | 1 (0.2) | 8 (1.0) |  |  |  |
| Paranoid Schizophrenia | 378 (28.5) | 161 (30.3) | 217 (27.2) |  |  |  |
| Hebephrenic schizophrenia | 19 (1.4) | 9 (1.7) | 10 (1.3) |  |  |  |
| Undifferentiated Schizophrenia | 883 (66.5) | 345 (65.0) | 538 (67.5) |  |  |  |
|  | Mean (SD) | Mean (SD) | Mean (SD) | *t* | *p* | SMD |
| Age (years) | 36.34 (12.73) | 36.89 (12.45) | 35.98 (12.90) | 1.28 | 0.20 | 0.071 |
| Length of Hospital Stay (days) | 44.39 (43.89) | 45.20 (30.89) | 43.85 (50.75) | 0.60 | 0.55 | 0.032 |
| Initial PANSS Score | 64.39 (20.27) | 63.67 (20.44) | 64.87 (20.16) | -1.06 | 0.29 | 0.059 |
| Final PANSS Score | 47.33 (13.90) | 45.72 (13.19) | 48.39 (14.26) | -3.46 | <0.001 | 0.194 |
| PANSS Reduction Score | 17.06 (17.45) | 17.93 (18.02) | 16.49 (17.06) | 1.45 | 0.15 | 0.082 |
| PANSS Reduction Percentage (%) | 22.66 (24.47) | 24.30 (22.11) | 21.59 (25.87) | 2.03 | 0.043 | 0.113 |
| Initial PANSS -Positive Score | 10.53 (4.38) | 10.43 (4.38) | 10.59 (4.38) | -0.68 | 0.49 | 0.038 |
| Final PANSS -Positive Score | 6.67 (2.44) | 6.50 (2.24) | 6.79 (2.57) | -2.19 | 0.029 | 0.121 |
| PANSS -Positive Reduction Score | 3.86 (4.14) | 3.93 (4.18) | 3.81 (4.12) | 0.53 | 0.60 | 0.030 |
| PANSS -Positive Reduction Percentage (%) | 29.30 (27.70) | 30.03 (27.28) | 28.81 (27.98) | 0.79 | 0.43 | 0.044 |
| Initial PANSS -Negative Score | 15.07 (7.43) | 14.91 (7.34) | 15.17 (7.49) | -0.63 | 0.53 | 0.035 |
| Final PANSS -Negative Score | 12.06 (5.83) | 11.55 (5.63) | 12.40 (5.93) | -2.65 | 0.008 | 0.148 |
| PANSS -Negative Reduction Score | 3.01 (5.98) | 3.36 (5.99) | 2.77 (5.96) | 1.77 | 0.078 | 0.099 |
| PANSS -Negative Reduction Percentage (%) | 11.48 (40.35) | 15.22 (32.11) | 8.98 (44.87) | 2.95 | 0.003 | 0.160 |
| Initial PANSS -General Score | 28.68 (8.99) | 28.39 (9.09) | 28.88 (8.92) | -0.97 | 0.33 | 0.055 |
| Final PANSS -General Score | 21.95 (6.22) | 21.25 (5.85) | 22.41 (6.42) | -3.40 | <0.001 | 0.189 |
| PANSS -General Reduction Score | 6.73 (8.03) | 7.13 (8.28) | 6.47 (7.85) | 1.47 | 0.14 | 0.083 |
| PANSS -General Reduction Percentage (%) | 18.78 (33.75) | 21.13 (21.81) | 17.21 (39.70) | 2.31 | 0.021 | 0.122 |
| Initial HAMD Score | 4.65 (6.72) | 4.67 (6.56) | 4.65 (6.86) | 0.03 | 0.98 | 0.003 |
| Final HAMD Score | 2.10 (3.68) | 2.27 (3.87) | 1.98 (3.55) | 0.68 | 0.49 | 0.078 |
| HAMD Reduction Score | 2.58 (5.46) | 2.43 (4.98) | 2.69 (5.78) | -0.43 | 0.67 | 0.049 |
| HAMD Reduction Percentage (%) | 40.19 (104.47) | 45.64 (57.51) | 36.05 (129.33) | 0.70 | 0.49 | 0.096 |
| Initial HAMA Score | 4.03 (5.54) | 3.58 (5.07) | 4.34 (5.83) | -2.42 | 0.016 | 0.140 |
| Final HAMA Score | 1.54 (3.17) | 1.25 (2.56) | 1.74 (3.51) | -2.81 | 0.005 | 0.160 |
| HAMA Reduction Score | 2.50 (4.81) | 2.32 (4.51) | 2.63 (5.01) | -1.10 | 0.27 | 0.064 |
| HAMA Reduction Percentage (%) | 61.72 (59.85) | 65.34 (52.99) | 59.42 (63.76) | 1.33 | 0.18 | 0.101 |
| Hospitalization Cost excluding the PGx testing cost(CNY) | 54464.17 (38867.76) | 56745.51 (32055.59) | 52944.23 (42761.03) | 1.85 | 0.065 | 0.101 |
| Hospitalization Cost (CNY) | 55919.35 (38989.19) | 60384.84 (32041.11) | 52944.23 (42761.03) | 3.62 | <0.001 | 0.197 |
| Frequency of outpatient visits within one year after discharge | 6.16 (6.54) | 6.31 (6.62) | 6.05 (6.48) | 0.69 | 0.49 | 0.039 |
| Frequency of readmissions within one year after discharge | 0.35 (0.82) | 0.42 (0.92) | 0.31 (0.74) | 2.31 | 0.021 | 0.132 |

*Fisher's Exact Test

**Table 2. Demographic and Clinical Characteristics of Patients with MDD**

| Variable | Overall  (n=1143) | PGx Testing Performed | | Univariable Analysis | | |
| --- | --- | --- | --- | --- | --- | --- |
|  |  | Yes (n=515) | No (n=628) |  |  |  |
|  | n (%) | n (%) | n (%) | Chi-square | *p* | SMD |
| Gender = Female | 888 (77.7) | 410 (79.6) | 478 (76.1) | 1.80 | 0.18 | 0.084 |
| Medical Insurance = Yes | 521 (45.6) | 250 (48.5) | 271 (43.2) | 3.10 | 0.078 | 0.108 |
| MECT Performed = Yes | 551 (48.2) | 250 (48.5) | 301 (47.9) | 0.02 | 0.88 | 0.012 |
| Primary Clinical Diagnosis |  |  |  | — | 0.28* | 0.222 |
| Recurrent depressive disorder | 14 (1.2) | 3 (0.6) | 11 (1.8) |  |  |  |
| Recurrent depressive disorder, current episode severe with psychotic symptoms | 207 (18.1) | 86 (16.7) | 121 (19.3) |  |  |  |
| Recurrent depressive disorder, current episode mild with somatic symptoms | 6 (0.5) | 4 (0.8) | 2 (0.3) |  |  |  |
| Recurrent depressive disorder, current episode moderate with somatic symptoms | 101 (8.8) | 51 (9.9) | 50 (8.0) |  |  |  |
| Recurrent depressive disorder, current episode severe without psychotic symptoms | 690 (60.4) | 316 (61.4) | 374 (59.6) |  |  |  |
| Recurrent depressive disorder, current episode mild without somatic symptoms | 2 (0.2) | 0 (0.0) | 2 (0.3) |  |  |  |
| Recurrent depressive disorder, current episode moderate without somatic symptoms | 44 (3.8) | 25 (4.9) | 19 (3.0) |  |  |  |
| Recurrent depressive disorder, currently in remission | 3 (0.3) | 1 (0.2) | 2 (0.3) |  |  |  |
| Recurrent depressive disorder, current episode mild | 14 (1.2) | 6 (1.2) | 8 (1.3) |  |  |  |
| Recurrent depressive disorder, current episode moderate | 42 (3.7) | 17 (3.3) | 25 (4.0) |  |  |  |
| Mixed anxiety and depressive disorder | 15 (1.3) | 5 (1.0) | 10 (1.6) |  |  |  |
| Organic depressive disorder | 5 (0.4) | 1 (0.2) | 4 (0.6) |  |  |  |
|  | Mean (SD) | Mean (SD) | Mean (SD) | *t* | *p* | SMD |
| Age (years) | 39.49 (18.28) | 39.72 (18.58) | 39.29 (18.05) | 0.39 | 0.69 | 0.023 |
| Length of Hospital Stay (days) | 31.64 (23.69) | 33.98 (21.40) | 29.73 (25.28) | 3.08 | 0.002 | 0.182 |
| Initial HAMD Score | 21.65 (10.75) | 22.78 (10.48) | 20.73 (10.89) | 3.23 | 0.001 | 0.192 |
| Final HAMD Score | 7.40 (7.73) | 7.47 (8.04) | 7.34 (7.47) | 0.29 | 0.77 | 0.017 |
| HAMD Reduction Score | 14.28 (11.22) | 15.35 (11.01) | 13.41 (11.32) | 2.91 | 0.004 | 0.173 |
| HAMD Reduction Percentage (%) | 59.60 (58.06) | 63.95 (33.77) | 56.04 (71.94) | 2.42 | 0.016 | 0.141 |
| Initial HAMA Score | 14.98 (9.19) | 15.76 (9.05) | 14.33 (9.26) | 2.62 | 0.009 | 0.156 |
| Final HAMA Score | 5.41 (6.08) | 5.43 (6.13) | 5.40 (6.04) | 0.08 | 0.94 | 0.005 |
| HAMA Reduction Score | 9.59 (8.87) | 10.39 (9.18) | 8.93 (8.56) | 2.74 | 0.006 | 0.164 |
| HAMA Reduction Percentage (%) | 57.75 (47.05) | 59.25 (50.09) | 56.50 (44.38) | 0.95 | 0.34 | 0.058 |
| Hospitalization Cost excluding the PGx testing cost(CNY) | 43083.30 (28521.33) | 47879.87 (30596.43) | 39149.81 (26073.03) | 5.13 | <0.001 | 0.307 |
| Hospitalization Cost (CNY) | 45001.52 (28947.45) | 52137.19 (30653.75) | 39149.81 (26073.03) | 7.62 | <0.001 | 0.456 |
| Frequency of outpatient visits within one year after discharge | 8.51 (7.78) | 9.25 (7.84) | 7.90 (7.68) | 2.91 | 0.004 | 0.173 |
| Frequency of readmissions within one year after discharge | 0.40 (0.97) | 0.50 (1.12) | 0.31 (0.82) | 3.25 | 0.001 | 0.196 |

**Table 3. Demographic and Clinical Characteristics of Patients with BD**

| Variable | Overall  (n=1471) | PGx Testing Performed | | Univariable Analysis | | |
| --- | --- | --- | --- | --- | --- | --- |
|  |  | Yes (n=501) | No (n=970) |  |  |  |
|  | n (%) | n (%) | n (%) | Chi-square | *p* | SMD |
| Gender = Female | 1010 (68.7) | 381 (76.0) | 629 (64.8) | 18.75 | <0.001 | 0.247 |
| Medical Insurance = Yes | 781 (53.1) | 307 (61.3) | 474 (48.9) | 19.94 | <0.001 | 0.251 |
| MECT Performed = Yes | 948 (64.4) | 338 (67.5) | 610 (62.9) | 2.83 | 0.093 | 0.096 |
| Primary Clinical Diagnosis |  |  |  | — | 0.33* | 0.201 |
| Bipolar affective disorder | 22 (1.5) | 7 (1.4) | 15 (1.5) |  |  |  |
| Bipolar affective disorder, current episode manic with psychotic symptoms | 451 (30.7) | 157 (31.3) | 294 (30.3) |  |  |  |
| Bipolar affective disorder, current episode severe depression with psychotic symptoms | 83 (5.6) | 23 (4.6) | 60 (6.2) |  |  |  |
| Bipolar affective disorder, current episode moderate depression with somatic symptoms | 19 (1.3) | 5 (1.0) | 14 (1.4) |  |  |  |
| Bipolar affective disorder, current episode manic without psychotic symptoms | 440 (29.9) | 137 (27.3) | 303 (31.2) |  |  |  |
| Bipolar affective disorder, current episode severe depressive without psychotic symptoms | 201 (13.7) | 79 (15.8) | 122 (12.6) |  |  |  |
| Bipolar affective disorder, current episode mild depression without somatic symptoms | 1 (0.1) | 0 (0.0) | 1 (0.1) |  |  |  |
| Bipolar affective disorder, current episode moderate depression without somatic symptoms | 16 (1.1) | 6 (1.2) | 10 (1.0) |  |  |  |
| Bipolar affective disorder, currently in remission | 6 (0.4) | 1 (0.2) | 5 (0.5) |  |  |  |
| Bipolar affective disorder, current episode mixed | 216 (14.7) | 82 (16.4) | 134 (13.8) |  |  |  |
| Bipolar affective disorder, current episode hypomanic | 10 (0.7) | 1 (0.2) | 9 (0.9) |  |  |  |
| Bipolar affective disorder, current episode moderate depression | 6 (0.4) | 3 (0.6) | 3 (0.3) |  |  |  |
|  | Mean (SD) | Mean (SD) | Mean (SD) | *t* | *p* | SMD |
| Age (years) | 35.38 (13.96) | 36.03 (13.50) | 35.04 (14.18) | 1.31 | 0.19 | 0.072 |
| Length of Hospital Stay (days) | 35.10 (21.73) | 35.66 (18.44) | 34.81 (23.25) | 0.76 | 0.45 | 0.04 |
| Initial YMRS Score | 15.21 (12.37) | 15.32 (12.67) | 15.15 (12.21) | 0.24 | 0.81 | 0.013 |
| Final YMRS Score | 3.36 (5.59) | 3.18 (5.57) | 3.46 (5.59) | -0.91 | 0.36 | 0.051 |
| YMRS Reduction Score | 11.83 (11.89) | 12.09 (12.14) | 11.70 (11.76) | 0.60 | 0.55 | 0.033 |
| YMRS Reduction Percentage (%) | 70.55 (49.55) | 72.51 (53.04) | 69.56 (47.70) | 0.95 | 0.34 | 0.058 |
| Initial HAMD Score | 10.39 (10.92) | 11.75 (11.99) | 9.70 (10.26) | 3.24 | 0.001 | 0.184 |
| Final HAMD Score | 3.28 (5.12) | 3.46 (5.52) | 3.19 (4.90) | 0.91 | 0.36 | 0.051 |
| HAMD Reduction Score | 7.10 (9.35) | 8.27 (10.32) | 6.50 (8.76) | 3.25 | 0.001 | 0.185 |
| HAMD Reduction Percentage (%) | 59.13 (64.69) | 64.33 (49.77) | 56.30 (71.42) | 2.17 | 0.030 | 0.130 |
| Initial HAMA Score | 7.11 (8.23) | 8.02 (9.03) | 6.65 (7.75) | 2.89 | 0.004 | 0.163 |
| Final HAMA Score | 2.48 (4.15) | 2.80 (4.57) | 2.32 (3.90) | 2.02 | 0.044 | 0.114 |
| HAMA Reduction Score | 4.59 (6.97) | 5.20 (7.54) | 4.26 (6.63) | 2.34 | 0.019 | 0.132 |
| HAMA Reduction Percentage (%) | 58.20 (62.20) | 58.96 (59.26) | 57.78 (63.81) | 0.29 | 0.77 | 0.019 |
| Hospitalization Cost excluding the PGx testing cost(CNY) | 47046.85 (25119.65) | 48960.99 (21687.36) | 46058.21 (26677.20) | 2.24 | 0.025 | 0.119 |
| Hospitalization Cost (CNY) | 48520.87 (25310.01) | 53288.89 (21669.95) | 46058.21 (26677.20) | 5.59 | <0.001 | 0.298 |
| Frequency of outpatient visits within one year after discharge | 7.24 (6.50) | 8.41 (6.99) | 6.64 (6.15) | 4.80 | <0.001 | 0.269 |
| Frequency of readmissions within one year after discharge | 0.38 (0.85) | 0.50 (0.95) | 0.32 (0.78) | 3.57 | <0.001 | 0.203 |

**Table 4. Linear Mixed Model Analysis of PANSS Score Changes with PGX Treatment in patients with SCZ**

| Variable | Estimate | UL | LL | statistic | *p* |
| --- | --- | --- | --- | --- | --- |
| Weeks of Hospitalization | -0.98 | -1.11 | -0.85 | -14.60 | <0.001 |
| PGx Testing Performed (Yes vs. No) | 0.95 | -0.94 | 2.84 | 0.99 | 0.32 |
| Gender (Female vs. Male) | -0.67 | -2.44 | 1.1 | -0.75 | 0.46 |
| Age (years) | 0.02 | -0.05 | 0.08 | 0.52 | 0.60 |
| MECT Performed (Yes vs. No) | 3.03 | 1.32 | 4.74 | 3.47 | <0.001 |
| Weeks of Hospitalization × PGx Testing Performed (Yes vs. No) | -0.86 | -1.10 | -0.61 | -6.85 | <0.001 |

**Table 5. Linear Mixed Model Analysis of HAMD Score Changes with PGX Treatment in patients with MDD**

| Variable | Estimate | UL | LL | statistic | *p* |
| --- | --- | --- | --- | --- | --- |
| Weeks of Hospitalization | -1.76 | -1.92 | -1.60 | -22.00 | <0.001 |
| PGx Testing Performed (Yes vs. No) | 2.77 | 1.71 | 3.84 | 5.12 | <0.001 |
| Gender (Female vs. Male) | 0.74 | -0.36 | 1.84 | 1.31 | 0.19 |
| Age (years) | 0.02 | -0.01 | 0.05 | 1.57 | 0.12 |
| MECT Performed (Yes vs. No) | 2.91 | 1.99 | 3.82 | 6.24 | <0.001 |
| Weeks of Hospitalization × PGx Testing Performed (Yes vs. No) | -0.49 | -0.73 | -0.26 | -4.13 | <0.001 |

**Table 6. Linear Mixed Model Analysis of HAMA Score Changes with PGX Treatment in patients with MDD**

| Variable | Estimate | UL | LL | statistic | *p* |
| --- | --- | --- | --- | --- | --- |
| Weeks of Hospitalization | -1.21 | -1.33 | -1.08 | -19.20 | <0.001 |
| PGx Testing Performed (Yes vs. No) | 1.79 | 0.91 | 2.70 | 3.99 | <0.001 |
| Gender (Female vs. Male) | 1.09 | 0.16 | 2.02 | 2.31 | 0.021 |
| Age (years) | 0.05 | 0.03 | 0.08 | 4.98 | <0.001 |
| MECT Performed (Yes vs. No) | 0.61 | -0.16 | 1.38 | 1.55 | 0.12 |
| Weeks of Hospitalization × PGx Testing Performed (Yes vs. No) | -0.32 | -0.51 | -0.14 | -3.47 | <0.001 |

**Table 7. Linear Mixed Model Analysis of YMRS Score Changes with PGX Treatment in patients with BD**

| Variable | Estimate | UL | LL | statistic | *p* |
| --- | --- | --- | --- | --- | --- |
| Weeks of Hospitalization | -1.52 | -1.64 | -1.40 | -24.20 | <0.001 |
| PGx Testing Performed (Yes vs. No) | 0.59 | -0.43 | 1.60 | 1.13 | 0.26 |
| Gender (Female vs. Male) | -0.39 | -1.26 | 0.48 | -0.88 | 0.38 |
| Age (years) | 0.03 | 0.001 | 0.06 | 2.00 | 0.046 |
| MECT Performed (Yes vs. No) | 2.27 | 1.43 | 3.12 | 5.27 | <0.001 |
| Weeks of Hospitalization × PGx Testing Performed (Yes vs. No) | -0.25 | -0.48 | -0.03 | -2.24 | 0.025 |

**Table 8. Linear Mixed Model Analysis of HAMD Score Changes with PGX Treatment in patients with BD**

| Variable | Estimate | UL | LL | statistic | *p* |
| --- | --- | --- | --- | --- | --- |
| Weeks of Hospitalization | -0.95 | -1.05 | -0.86 | -19.20 | <0.001 |
| PGx Testing Performed (Yes vs. No) | 2.16 | 1.27 | 3.04 | 4.78 | <0.001 |
| Gender (Female vs. Male) | 2.00 | 1.22 | 2.79 | 5.00 | <0.001 |
| Age (years) | -0.03 | -0.06 | -0.004 | -2.28 | 0.023 |
| MECT Performed (Yes vs. No) | -0.10 | -0.89 | 0.68 | -0.26 | 0.80 |
| Weeks of Hospitalization × PGx Testing Performed (Yes vs. No) | -0.39 | -0.56 | -0.22 | -4.54 | <0.001 |

**Table 9. Age-Stratified Analysis of Scale Score Reductions in Three Psychiatric Disorders**

| Disorder | Scale | Age | Variable | PGx Group | TAU Group | *p* |
| --- | --- | --- | --- | --- | --- | --- |
| SCZ | PANSS | ＜18 | Reduction Score | 15.76 | 18.16 | 0.6839 |
|  |  |  | Reduction Percentage (%) | 24.05 | 23.06 | 0.8901 |
|  |  | 18-64 | Reduction Score | 18.44 | 16.72 | 0.1070 |
|  |  |  | Reduction Percentage (%) | 25.64 | 22.39 | 0.0303 |
|  |  | ＞65 | Reduction Score | 22.33 | 13.77 | 0.2500 |
|  |  |  | Reduction Percentage (%) | 35.16 | 21.20 | 0.1606 |
| MDD | HAMD | ＜18 | Reduction Score | 12.22 | 10.91 | 0.4009 |
|  |  |  | Reduction Percentage (%) | 54.83 | 54.44 | 0.9513 |
|  |  | 18-64 | Reduction Score | 15.94 | 13.86 | 0.0082 |
|  |  |  | Reduction Percentage (%) | 65.93 | 57.66 | 0.0432 |
|  |  | ＞65 | Reduction Score | 14.93 | 12.76 | 0.2341 |
|  |  |  | Reduction Percentage (%) | 64.54 | 49.41 | 0.2126 |
|  | HAMA | ＜18 | Reduction Score | 8.04 | 6.47 | 0.1650 |
|  |  |  | Reduction Percentage (%) | 55.20 | 49.43 | 0.3780 |
|  |  | 18-64 | Reduction Score | 10.49 | 9.16 | 0.0327 |
|  |  |  | Reduction Percentage (%) | 60.09 | 58.58 | 0.6456 |
|  |  | ＞65 | Reduction Score | 12.08 | 9.61 | 0.1309 |
|  |  |  | Reduction Percentage (%) | 61.23 | 52.22 | 0.3707 |
| BD | YUNG | ＜18 | Reduction Score | 8.88 | 6.10 | 0.2053 |
|  |  |  | Reduction Percentage (%) | 82.31 | 55.32 | 0.0630 |
|  |  | 18-64 | Reduction Score | 11.03 | 11.34 | 0.6369 |
|  |  |  | Reduction Percentage (%) | 72.05 | 70.46 | 0.6094 |
|  |  | ＞65 | Reduction Score | 11.54 | 10.17 | 0.7420 |
|  |  |  | Reduction Percentage (%) | 88.31 | 80.74 | 0.4583 |
|  | HAMD | ＜18 | Reduction Score | 8.21 | 9.56 | 0.5519 |
|  |  |  | Reduction Percentage (%) | 45.97 | 53.34 | 0.6244 |
|  |  | 18-64 | Reduction Score | 8.62 | 6.47 | ＜0.001 |
|  |  |  | Reduction Percentage (%) | 66.03 | 57.31 | 0.0282 |
|  |  | ＞65 | Reduction Score | 12.08 | 6.51 | 0.0945 |
|  |  |  | Reduction Percentage (%) | 72.69 | 53.44 | 0.2502 |
